# Posttraumatic stress, visual working memory, and visual imagery in military personnel

**DOI:** 10.1007/s12144-023-04338-1

**Published:** 2023-02-21

**Authors:** Brenton Russell, Alexander J. Mussap

**Affiliations:** School of Psychology, Deakin University, 221 Burwood Highway, 3125 Melbourne, Australia

**Keywords:** Posttraumatic stress, Military personnel, Working memory, N-back, Visual imagery

## Abstract

Posttraumatic stress disorder (PTSD) is characterized by disruptions to cognitive functioning. Two studies were conducted to examine the relevance of military-related PTSD to two cognitive functions – visual working memory and visual imagery. Participants were military personnel who reported their PTSD diagnosis history and completed a self-administered screening tool for PTSD, the PTSD Checklist – Military Version. In Study 1, 138 personnel also completed a memory span task and a 2-back task using colored words in which Stroop interference was introduced via the semantic content of the words. In Study 2, a separate group of 211 personnel completed measures of perceived imagery vividness and spontaneous use of visual imagery. Interference effects on working memory in PTSD-diagnosed military personnel were not replicated. However, ANCOVA and structural equation modelling revealed that PTSD-intrusions were associated with poorer working memory whereas PTSD-arousal was associated with spontaneous use of visual imagery. We interpret these results as evidence that intrusive flashbacks disrupt working memory performance not by limiting memory capacity nor by interfering directly with memory functions such as inhibition, but by adding internal noise in the form of task-irrelevant memories and emotions. Visual imagery appears to be unrelated to these flashbacks but with arousal symptoms of PTSD, perhaps in the form of flashforwards about feared/anticipated threats.

## Introduction

Posttraumatic stress disorder (PTSD) is a maladaptive emotional and cognitive response to a traumatic event characterized by intrusions in the form of unwanted memories and emotions of the event, avoidance, negative cognition and mood, and arousal, hypervigilance, and anticipated fear (American Psychiatric Association, 2022). The condition is prevalent and severe in military personnel (Gerber et al., 2018; Reger et al., 2019).

PTSD can be caused by exposure to a range of hazards encountered during military service (Brownlow et al., 2018), including actual or threatened physical harm (Kessler et al., 2017), witnessing the depravities of war (Nordstrand et al., 2019; VanBergen et al., 2020), and ‘moral injury’ caused by violations of one’s moral code or professional ethics (Hall et al., 2022; Litz et al., 2009). Military personnel who do not meet the diagnostic criteria for PTSD can still develop subclinical levels of posttraumatic stress symptoms (PTSS) following exposure to these hazards (Erickson et al., 2013).

PTSD/PTSS is associated with alterations to cognitive-affective functioning particularly in relation to attention and memory for trauma-related information (Blanchette & Caparos, 2016). These alterations are thought to be both manifestations of PTSD symptoms as well as factors that undermine executive functioning in such a manner as to exacerbate and maintain symptoms of the disorder (Daneshvar et al., 2021). They are thought to contribute to the risk of psychological difficulties experienced by military personnel, including drug and alcohol dependence, aggression, self-harm, and widespread disruption to personal and professional life (Armenta et al., 2019; Knobloch et al., 2022).

### Working memory and affective control in PTSD/PTSS

Working memory (WM) is a low-capacity storage system consisting of (i) a central executive that directs attention to sensory inputs, (ii) short-term memory stores in the form of a phonological loop and a visuospatial sketchpad, with (iii) access to a long-term memory store (Baddeley, 1986, 2002). WM is responsive to changing task demands, evaluates incoming information in terms of its relevance to the task, inhibits information deemed to be irrelevant, and selects and maintains relevant information for use by higher-level cognitive systems (Shipstead et al., 2012). Because of its role in prioritizing and manipulating information in real time, WM is thought to underpin numerous important capabilities including learning, problem solving, reasoning, remembering and also forgetting (Nejati et al., 2018).

Affective content appears to tax WM regardless of its valence. This is thought to be because emotionally salient information in real-world situations often signals the presence of potential hazards or rewards (Rączy & Orzechowski, 2021). People diagnosed with affect-related disorders, including PTSD (Larsen et al., 2019), exhibit additional WM deficiencies (Blanchette & Caparos, 2016) that point to pronounced difficulties in managing affective content (Larsen et al., 2019). This has been demonstrated across a variety of WM tasks (simple capacity, complex capacity, n-back, Stroop), across sensory modalities (visual and auditory), distractor type (words, images of inanimate objects, facial expressions), task relevance (and if relevant, congruous or incongruous), and valence (neutral, negative-emotional, positive-emotional) (see review by Larsen et al., 2019). However, no coherent explanation for these difficulties exists (Schweizer et al., 2020), presumably because the putative central executive system of WM is poorly understood as is the nature of the relationship between affect and attention in the context of this system.

### Emotional Stroop interference

Emotional-Stroop tests provide a way of measuring the effects of emotional interference on memory tasks. In the original Stroop, the ability to name colored letters deteriorates when the letters are arranged into a word that spells an incongruous color (Ben-Haim et al., 2016). There is general agreement that this task taps into affective control processes that inhibit task-irrelevant information (Larsen et al., 2019). When color names are replaced with emotional words, Stroop performance deteriorates further (Ben-Haim et al., 2016). The magnitude of this effect reflects a participant’s underlying concerns. For example, a person diagnosed with depression is usually more sensitive to distractors in the form of negative mood words (Schweizer et al., 2020). The effect can also be induced experimentally using mood induction (Ribeiro et al., 2019). Research shows that people diagnosed with PTSD are more likely than controls to have difficulty with emotional Stroop tasks, particularly in the presence of depression and anxiety-related distractors (Mathew et al., 2022).

The contextual nature of PTSD complicates what constitutes a salient negative emotional distractor because an otherwise innocuous stimulus can be distressing if it happens to be associated with the original traumatic event. Perhaps for this reason, most research in the area has opted for generic emotional stimuli (rather than trauma-specific ones) whose valence and salience are not dependent on context or the personal history of the respondent. Given this omission, we suggest that research into PTSD/PTSS in the military also include distractors that are specifically meaningful to military personnel (Larsen et al., 2019). In the following section we explain how emotional and military-specific content may be introduced into memory tasks to measure the functioning and resilience of various components of memory.

### Memory span and WM capacity

Because WM is a multi-component system that interacts with other cognitive processes such as short-term memory (STM) and attention (Baddeley, 2002), effort needs to be made to ensure that what is being measured – and the deficits being observed – can be attributable to WM.

Simple span tests measure test recall for the order of a sequence of stimuli (e.g., letters or numbers) as a function of their overall length or duration. Performance varies depending on stimulus type but averages at around seven items and an overall duration of 15 to 30 s (Atkinson & Shiffrin, 1971). These tasks are considered measures of generic temporary information storage reflecting the efforts of diverse cognitive systems (Kane & Engle, 2003). Performance in these tasks is thought to be relevant to WM only indirectly in the sense that WM processes are thought to be able to call upon information stored in STM when their own capacity is exceeded (Baddeley, 1986). STM deficiencies have indeed been observed in relation to both PTSS and PTSD under high cognitive load (Judah et al., 2018).

N-back tasks also present participants with a sequence of stimuli, but in this case participants are asked to provide a response to each stimulus (e.g., “same/different”) in relation to the stimulus that occurred ‘N’ steps earlier in the sequence. The task requires that participants select one stimulus from storage and make a decision about it using the current stimulus as a reference. The ability to do so is thought to reflect WM functioning in relation to selecting, inhibiting, and updating stored content (Berger et al., 2017). N-back performance is consistently worse in people diagnosed with PTSD and in relation to PTSS over a broad range of stimulus types and response criteria (Nejati et al., 2018).

### Visual imagery and PTSD/PTSS

The visuospatial sketchpad manages visual information – seen images that are retained, images recalled from long-term memory (LTM), or made-up images that are imagined – for use by the central executive of WM (Baddeley, 2002). There is experimental evidence that the sketchpad shares processes with visual imagery (Baddeley & Andrade, 2000). For example, self-reported visual imagery vividness is related to visual WM capacity as measured by the ability to make retrospective spatial comparisons of stored visual images (Keogh & Pearson, 2014). Vividness of visual imagery is also associated with PTSS/PTSD, particularly in relation to intrusions in the form of visual flashbacks (Bryant & Harvey, 1996), with neuroimaging evidence suggesting that this reflects a tendency towards visually re-experiencing rather than controlling autobiographical memories of trauma (Thome et al., 2020). Trauma can also induce anticipatory and imagined fear that can take the form of vivid and detailed future-oriented mental images or ‘flashforwards’ (Holmes et al., 2007; Rachman & de Silva, 1978). For instance, people with depression or PTSD may suffer from future-oriented images that represent an elaboration of a specific personal memory, like a person nursing a sick relative imagining that relative deteriorating further or dying, or a person who had a stroke imagining suffering another one (Reynolds & Brewin, 1998). In one of the few studies in the area with pre-trauma measures and time-order data, (non-PTSD) participants completed a subjective imagery vividness questionnaire prior to being exposed to a distressing video (Morina et al., 2013). Vividness of imagery ratings were found to predict their self-reported vividness of subsequent intrusive memories of the video and emotional distress associated with these memories up to five days later. There is also a growing body of research supporting the therapeutic use of visual imagery in relation to PTSD-related negative mood (e.g., Kaimal et al., 2022).

In the present study, we addressed two issues concerning the link between PTSD and visual imagery. First, there is limited research into the question of whether PTSD symptom clusters, aside from intrusions and negative mood, are relevant to visual imagery. Second, the focus of this research has been on the phenomenological aspects of visual imagery, notably its subjective vividness (Mota et al., 2015). This focus has been criticised both because imagery experiences cannot be validated (Baddeley & Andrade, 2000), and because doing so ignores functionally-relevant components of imagery (Ahsen, 1995). Consider that even people diagnosed with aphantasia, who report impaired, diminished, or entirely absent visual imagery (Dance et al., 2022), can have normal visual memory span (Keogh et al., 2021). This suggests that the visuospatial sketchpad of WM can utilize visual information (e.g., retrieve, generate, and manipulate visual objects) without invoking visual experiences (Keogh et al., 2021). In the present study we addressed these issues by evaluating the relevance of PTSD to two components of visual imagery – visual imagery vividness and spontaneous use of visual imagery when carrying out everyday tasks.

### PTSD symptom clusters

According to the DSM 5 (American Psychiatric Association, 2022), a diagnosis of PTSD requires direct or indirect exposure to a severe stressor (the traumatic event) along with the following symptoms: Intrusive upsetting memories of the event and the emotions associated with it (symptom cluster B), avoidance of trauma-related thoughts, memories, or feelings (symptom cluster C), negative alterations in cognition and mood (symptom cluster D), and alterations in arousal and reactivity (symptom cluster E). The existence of these four different but highly correlated symptom clusters (we confirm these correlations in the present paper) complicates our understanding of the role of affect-related WM deficits in PTSS/PTSD. It makes it possible to explain WM deficits in PTSD in a variety of ways and makes it difficult to pit one explanation against the other. For example, the reason for the greater vulnerability to emotional distractors in PTSD may be that intrusive re-experiencing (symptom cluster B) interferes with processes that inhibit external noise or because the intrusions themselves constitute a source of internal noise that taxes these processes (Mathew et al., 2022); that the cognitive effort involved in avoidance strategies (symptom cluster C) when confronting trauma-related triggers interferes with normal cognitive functioning, including WM (Mathew et al., 2022); that higher baseline levels of negative cognition/mood (symptom cluster D) means that ambiguous or mildly negative content will be more likely to trigger negative responses (Boffa et al., 2018); and/or that emotional content may be primed and thus more psychologically impactful because of one’s pre-existing levels arousal/ hypervigilance (symptom cluster E) (Litz & Keane, 1989). Note that these explanations are not mutually exclusive, and it is possible that they all receive empirical support. Therefore, we argue for more research to determine how specific PTSD symptom clusters map onto to specific WM deficits (Mathew et al., 2022).

In terms of whether affective interference on WM in PTSD reflects vulnerabilities related to the operation or control of the visuospatial sketchpad, a reasonable possibility is that this might be the case for intrusions in the form of visual flashbacks (symptom cluster B) of autobiographical memories, images, and emotions relating to the traumatic event (Brewin et al., 1996; Bryant & Harvey, 1996). However, it is also reasonable to suggest that the involvement of visual imagery relates to a negative bias in cognition/mood (symptom cluster D) that prioritizes and enhances negative visual images making it more difficult to suppress them, or to a general hyperarousal (symptom cluster E) that increases the prevalence of visual images occurring. We argue that these explanations are testable by evaluating relationships between measures of visual imagery and measures of PTSD symptom clusters.

### General research aims

The literature review highlighted unresolved questions about the nature of WM functioning in military-related PTSS/PTSD. Despite both WM and PTSD being constructs that pose challenges for research in the area, several promising lines of enquiry were identified, including measuring vulnerability to emotional distractor information for different components of memory –simple memory span, WM, and visual imagery – and linking performance to specific PTSD symptom clusters. A case was also made for broadening the definition of ‘distractor’ in the PTSD context to include stimuli whose valence is determined by their association with the original traumatic event (in our study, these associations are military ones).

In response to this review, two studies were conducted to examine the relevance of military-related PTSD diagnosis and symptomatology to visual cognitive functioning. The aim of Study 1 was to examine relationships between PTSD and WM functioning in the presence of task-irrelevant distractors to determine which PTSD symptom clusters are relevant. The aim of Study 2 was to examine relationships between PTSD and visual imagery functioning – both vividness and spontaneous use of imagery – again in relation to PTSD symptom clusters.

## Study 1 – Working memory and PTSD symptomatology

Intrusive and emotionally distressing memories feature prominently in the PTSD symptoms of military personnel. In Study 1 we asked three questions about memory functioning in relation to affective content in this population: (i) Do military personnel diagnosed with PTSD exhibit impaired memory functioning and, if so, are their impairments reflective of basic limitations of memory store or limitations of WM? (ii) Are these limitations more pronounced when processing information that is emotionally salient and, if so, does this extend to information that is salient primarily through its association with military experiences and contexts? (iii) Which PTSD symptom clusters are most closely associated with memory functioning in military personnel?

To answer these questions, military personnel were asked to complete memory tasks involving sequences of colored words. The first task was a basic memory span test in which the length of the sequence of colored words was manipulated; the second was a 2-back WM task. Both tasks required that participants attend only to stimulus color, and both tasks included Stroop interference in the form of the content of the words – military, negative-emotional, and neutral (concrete nouns without emotional or military connotations). Participants’ PTSD diagnosis history and PTSD symptomatology were measured and used to predict their performance in these WM tasks as well as their ability to ignore Stroop interference while performing these tasks. The following hypotheses were tested:

H1: Memory performance will be worse in military personnel diagnosed with PTSD and negatively related to PTSS. These effects will be evident in terms of memory span (indicative of deficiencies in basic memory capacity), and/or 2-back performance (indicative of deficiencies in WM updating).

H2: Task-irrelevant information in the form of military-related or negative emotional content will impair memory performance in PTSD-diagnosed military personnel more so than in their non-PTSD peers.

H3: Differential associations between PTSD symptom clusters and memory performance will identify which characteristics of PTSD are most relevant to memory deficiencies in military personnel.

## Methods

### Participants

Participants were 138 military personnel between 21 and 84 years of age. Their characteristics are summarized in Table 1, showing them to be predominantly white Australian Defence Force Army personnel who occupied a combat role and deployed at least once. Prior to commencing the study, participants confirmed they had corrected-to-normal visual acuity and no known color vision deficits.

**Table 1:** Participant characteristics for Study 1 (N = 138) and Study 2 (N = 211)

		Study 1	Study 2				Study 1	Study 2
		N (%)	N (%)				N (%)	N (%)
Gender	Woman	25 (18.1)	39 (18.5)		Deployments (N)	M (SD)	2.88 (2.7)	2.94 (2.8)
	Man	110 (79.7)	167 (79.1)		Years of service	M (SD)	15.3 (10.5)	15.1 (9.7)
	Other	3 (2.2)	4 (1.9)		Currently serving		54 (39.1)	91 (43.1)
Ethnicity	Asian	4 (2.9)	7 (3.3)		Branch	Airforce	19 (13.8)	29 (13.7)
	Black	0	1 (0.5)			Army	103 (74.6)	157 (74.4)
	Indigenous	2 (1.4)	2 (0.9)			Navy	13 (9.4)	22 (10.4)
	Latino/Latina	0	5 (2.4)			Marines	1 (0.7)	2 (0.9)
	White	112 (81.2)	166 (78.7)		Military role(s)	Administration	33 (23.9)	51 (24.2)
	Multiethnic	5 (3.6)	6 (2.8)			Combat	85 (61.6)	138 (65.4)
	Other	7 (5.1)	7 (3.3)			Command	41 (29.7)	64 (30.33)
Nationality	Australia	120 (87.0)	167 (79.1)			Disaster Relief	29 (21.0)	42 (19.9)
	Ireland	1 (0.7)	0			Humanitarian/Aid	30 (21.7)	49 (23.3)
	Norway	0	1 (0.5)			Intelligence	15 (10.9)	20 (9.5)
	Singapore	1 (0.7)	3 (1.4)			Maintenance/Support	36 (26.1)	50 (23.7)
	South Africa	1 (0.7)	1 (0.5)			Peace-keeping	42 (30.4)	61 (28.9)
	UK	1 (0.7)	2 (0.9)			Staff-HQ	39 (28.3)	48 (22.8)
	USA	11 (8.0)	26 (12.3)			Training	65 (47.1)	99 (46.9)
Education	<High school	6 (4.3)	12 (5.7)		Deployed to…	Afghanistan	59 (42.8)	96 (45.5)
	High school	20 (14.5)	28 (13.3)			Iraq	48 (34.8)	56 (26.5)
	University/College	75 (54.3)	117 (55.7)			Timor-Leste	53 (38.4)	76 (36.0)
	Postgrad/Prof	37 (26.8)	53 (25.2)			Papua New Guinea	7 (5.1)	9 (4.3)
Income	<$30k	15 (11.0)	23 (10.9)			Balkans	3 (2.2)	5 (2.4)
	30-59k	20 (14.6)	34 (16.1)			…others (by region)		
	60-99k	63 (46.0)	87 (41.2)			Middle East	27 (19.6)	30 (14.2)
	100-149k	19 (13.9)	34 (16.1)			Asia	18 (13.0)	29 (13.7)
	> 150k	20 (14.6)	32 (15.2)			Oceania	24 (17.4)	30 (14.2)
PTSD	Diagnosis	60 (43.5)	97 (46.0)			Europe	1 (0.7)	5 (2.4)
PTSD	Treatment	58 (42.0)	89 (42.2)			Africa	9 (6.5)	14 (6.6)
Medications	M (SD)	2.37 (0.1)	2.32 (0.2)			Americas	8 (5.8)	8 (3.8)

### Procedure

The authors adhered to APA ethical standards in the recruitment and treatment of participants, and the project was approved by our institution’s Human Research Ethics Committee. Prior to commencing each phase of Study 1, participants were directed to an online plain language statement that summarized the ethical aspects of the study, including our general research aims, task demands including time commitment, potential risks to participants and measures in place to mitigate these risks (e.g., trigger warnings and links to mental health and veterans’ support services available in several countries), intended use of data, and processes to ensure privacy/confidentiality. The plain language statement concluded with a button that participants were invited to press to confirm that they understood and agreed with these conditions and consented to participate in the study.

Participants were recruited by the authors, one of whom is a veteran of the Australian Defence Force (ADF), and by assistant recruiters several of whom are also ADF veterans. We recruited through personal and professional networks, via LinkedIn™, and through moderated military-related social media sites. Participants were offered an honorarium in the form of entry to a raffle for one of three 100AUD e-gift cards.

The study was completed entirely online over two sessions a week apart via the Qualtrics™ survey system. The WM tasks were completed online using the participant’s own device and internet connection. All data were collected during Australia’s Covid-19 lockdowns of 2021. In the first session, participants answered demographic questions and questions about their service history (military role, rank, branch, and where/when they were deployed), history of PTSD diagnosis and treatment, use of prescription medicines, and visual functioning (participants were asked to confirm their visual acuity was corrected-to-normal and that they had no known color vision deficits). Participants then completed the following self-administered screen for PTSD:

### Materials

#### PTSD checklist – military version (PCL‑M)

Participants used 5-point Likert-type scales anchored from 0 (not at all) to 5 (extremely) to indicate the extent to which they had experienced 17 symptoms of PTSD in the previous month (Thome et al., 2020). Symptoms include examples of each of four PTSD criteria: Intrusions (B) (e.g., “*Repeated, disturbing dreams of a stressful military experience?*”), avoidance(C) (e.g., “*Avoiding activities or situations because they reminded you of a stressful military experience?*”), negative emotions/cognitions (D) (e.g., “*Loss of interest in activities that you used to enjoy?*”), and arousal (E) (e.g., “*Being “super-alert” or watchful or on guard?*”). Higher scores on the PCL-M indicate greater severity of these symptoms. The PCL-M has been validated for use with military personnel (Thome et al., 2020), and a confirmatory factor analysis (CFA) on the data from the present study supported the four diagnostic criteria for PTSD proposed by the DSM 5: χ^2^(112) = 206.62, *p* < .05, ^2^/df = 1.85, RMSEA = 0.079 [95% CI: 0.062-0.095], SRMR = 0.041, CFI = 0.96, and TLI = 0.95.

#### Color-word stimuli and presentation orders

The first session concluded either with a memory span task followed a week later by the 2-back task described below, or the 2-back task followed a week later by the memory span task. This one-week break was provided because both tasks were arduous. Stimuli were words in Arial caps typeface at 48 font size presented in the centre of the screen and colored either red (HTML color #e74c3c), green (HTML color #27ae60), or blue (HTML color #2980b9). The words used were military words, negative valence emotional words, and neutral valence concrete nouns grouped into 33 triads matched for number of syllables and, as far as possible, word length and frequency of common English usage (according to Hunston,^2002^). Examples of triads used in the study include: ARMY, ANGRY, ARTIST; BOMB, BAD, BUS; CONVOY, ANNOY, ALLOY; GUN, SAD, SUN; RAID, PAIN, MAID; SHOOT, SHOCK, SHOP; SOLDIER, DANGER, WAITER; TANK, HATE, PLATE; WAR, FEAR, CAR (the full list can be obtained from the authors). Words were selected from the word bank as a triad. For example, if a participant was randomly chosen to receive triad #1 as the first word in a sequence, then they would be presented with the three versions of that triad (i.e., ARMY, ANGRY, and ARTIST) as the first word in each sequence of this condition. This ensured that even as the color of words varied, the words themselves would be matched at the level of condition and position within the word sequence. Throughout the experiments, colors and word triads were selected pseudo-randomly with the following provisos: (i) The same color could not appear more than twice in a row within a sequence; (ii) the same word triad could not appear more than twice in the same sequence; and (iii) the triad-matched versions of same condition could not appear in the same block.

#### Memory span task

Memory span trials consisted of word sequences with each word presented in a pseudo-random color (red, green, or blue). Each word was programmed to appear for 500msec (although this would have been influenced by the refresh rate and internet speed of the device used by participants to complete the task online). For this reason, memory span performance (and 2-back performance) was measured exclusively in terms of correct performance rather than reaction time. Participants were presented with 45 word sequences consisting of 5 different word sequence lengths (4–8 words) by three word types (military, emotional, neutral – note that word type was held constant within a sequence) in random order. These were presented in three blocks of 15 sequences each with a participant-determined break provided between blocks. At the completion of each sequence, participants were presented with a 2D response grid with sequence position shown along rows (1st to n^th^) and color (red, green, blue) shown along columns. Participants used this grid to indicate the order of colors in each sequence. A correct response required that the participant correctly identify the full color sequence without error. No feedback was provided during actual trials, but before attempting the real trials participants were shown two examples of color sequences where the correct answer was revealed and explained. They then completed three practice trials consisting of randomly colored animal names of sequence length 4, 6, and 8 with correct/incorrect feedback provided after each sequence.

#### 2-back task

2-back trials consisted of sequences of ten words of the same type (military, emotional, or neutral) with each word presented in a pseudo-random color (red, green, or blue; see provisos listed in the subsection ‘color-word stimuli’). Immediately beneath each word were two buttons labelled YES and NO. When each word appeared, it would remain on the screen until the participant responded YES if it was the same color as the color of the word two steps earlier in the sequence, or NO if it was not. Once they responded, the next word in the sequence would appear. Participants completed a total of 18 2-back word sequences presented across three blocks of trials. The blocks each contained six 2-back sequences with each word type appearing twice. Participant-determined breaks were provided between each block. No feedback was provided, although prior to commencing, participants received three 2-back sequences of 10 animal words as practice, with feedback provided (along with an explanation and reminder of the task requirements if an incorrect response was given) after each sequence.

### Data scoring procedures

Survey items and memory tasks were subject to response validation to ensure that a response was provided to each item of a survey measure or each trial in a memory task before the participant could continue, thus ensuring that there were no missing values.

Due to our reliance on a self-report measure of military-related PTSD diagnosis history that could not be independently verified by a clinician, we validated responses against participants’ posttraumatic stress symptomatology as measured by the PCL-M. To this end, a binary logistic regression was conducted in which PTSD diagnosis (1 = not diagnosed; 2 = diagnosed) was regressed on the PCL-M subscales.

Correct responses to memory span trials for each condition (sequence length by word type) were summed across the three repeated blocks, giving a score for each condition between 0 and 3. Summed scores were plotted against sequence length (the x-axis) separately for each word type and were fit with a descending Weibull cumulative distribution. This fit returned the sequence length giving 50% correct performance, and this value was taken as the participant’s memory span for that word type (i.e., the higher the value the greater the memory span).

Responses to each stimulus in the 2-back sequences beyond position two in the sequence (the first two stimuli in a 2-back sequence must generate a NO response and were not counted for the purposes of these analyses) were pooled across the six repeats of the same word type and converted into a hit rate (H = proportion correct YES responses) and false alarm rate (F = proportion incorrect YES responses) for that word type. These were then converted into z scores from which ‘*d primes’* (one for each word type) could be calculated according to: *d’ = z(H) - z(F)* (Neil & Creelman, 2005). That is, the greater the *d prime* value, the better the participant’s 2-back performance.

### Data analysis

To test whether memory performance was impaired in military personnel diagnosed with PTSD compared to those without such a diagnosis, two 3 × 2 ANCOVAs were conducted separately on each measure of WM performance (memory span and 2-back) performed under three different types of interference (military, emotional, and neutral words), as a function of PTSD diagnosis history (with v. without a military-related PTSD diagnosis). Participant age was included as a covariate in these analyses for two reasons: (i) Participant age is directly related to time elapsed since index trauma, and previous longitudinal evidence supports a gradual (albeit heterogenous) reduction in PTSD symptoms over time (Lee et al., 2020), and (ii) there is evidence of age-related decline in a range of cognitive abilities, including visual WM performance (Tas et al., 2020).

**Fig. 1:**
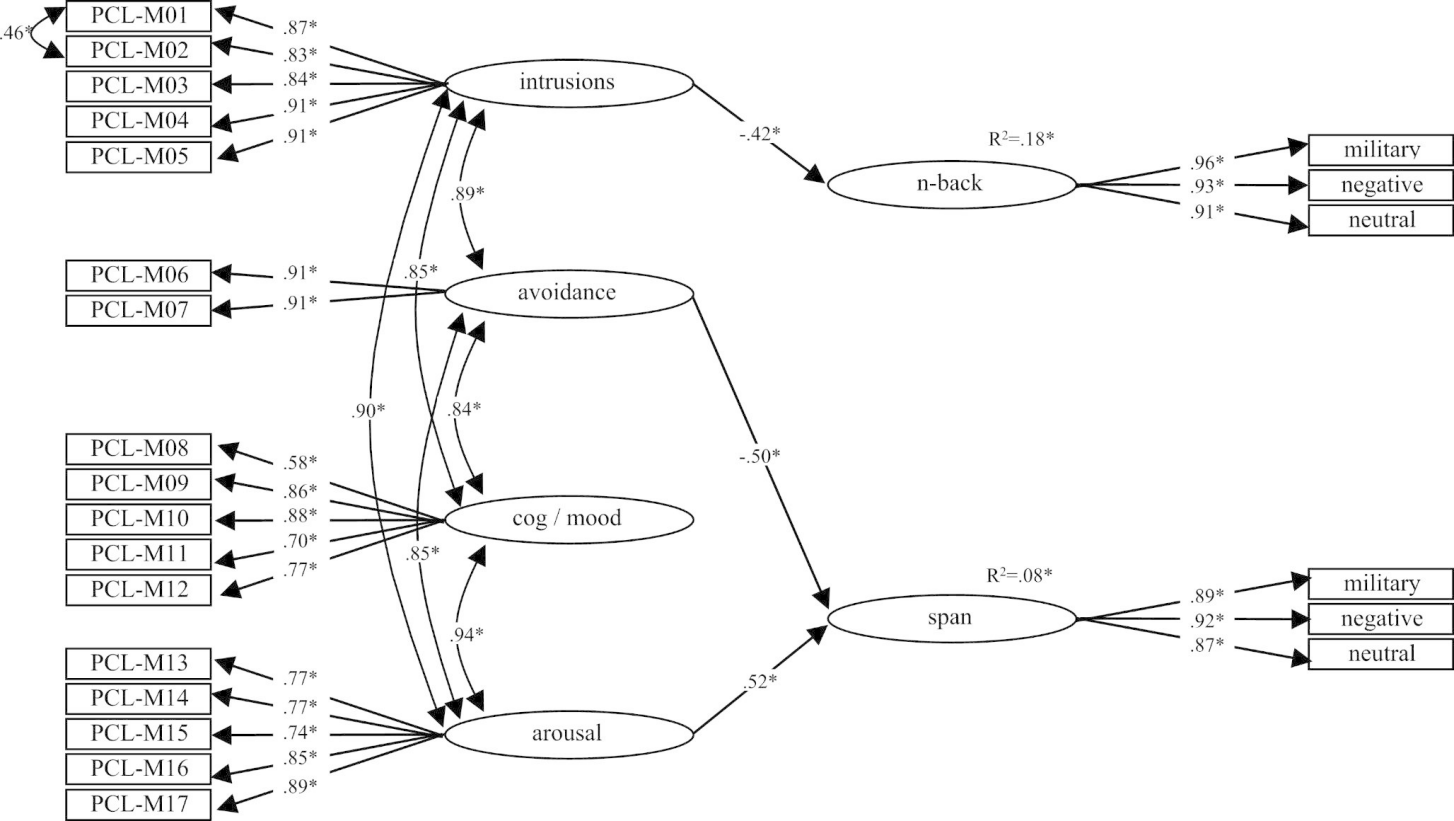
**Structural model for Study 1** (model optimized by removal of non-significant paths) with standardized statistics included. Model fit: χ^2^(220) = 309.08, *p* < .001; cχ^2^/df = 1.41; RMSEA = 0.054, 90% CI [0.039, 0.068]; SRMR = 0.045; CFI = 0.97, and TLI = 0.97; *p < .05; N = 138

To test associations between PTSS and WM performance, a structural model (Fig. 1) was developed to test for paths from PCL-M subscales to memory span and 2-back performance (this model originally included all possible paths from the PCL-M factors to the factors representing n-back and memory span performance). Prior to using this model it would be evaluated for fit in AMOS™ Version 25.0 (Amos Development Corporation, Meadville, PA, USA) using maximum likelihood estimation against the following standard fit criteria: χ^2^(df) non-significant; χ^2^/df < 5; root mean square error of approximation (RMSEA) < 0.08; standardized root mean square residual (SRMR < 0.08), comparative fit index (CFI) > 0.90, and Tucker-Lewis Index (TLI > 0.90) (Hu & Bentler, 1999).

## Results

Of the 157 participants who completed the PCL-M and the memory task of the first session, 138 returned a week later to complete the memory task of the second session, making the attrition rate 12%. Measures are summarised in Table 2 along with bivariate correlations. The table confirms the presence of adequate normality in these measures along with high intercorrelations between the subscales of the PCL-M.

**Table 2:** Descriptives and bivariate correlations

																		non-PTSD	PTSD
	Study 1 (n = 138)	1	2	3	4	5	6	7	8	9	10	11	min	max	α	Sk	K	M (SD)	M (SD)
1	Age	-	0.19*	0.10	0.15	0.18*	− 0.07	− 0.06	0.02	− 0.07	− 0.03	− 0.04	22	75	-	1.80	− 0.05	43.06 (10.8)	49.03 (11.5)
2	PCL-M Intrusions		-	0.83**	0.79**	0.82**	0.01	0.09	0.05	− 0.40**	− 0.39**	− 0.36**	0	20	0.94	2.14	-2.78	3.91 (4.3)	11.18 (4.9)
3	PCL-M Avoidance			-	0.78**	0.77**	− 0.06	0.04	− 0.06	− 0.36**	− 0.32**	− 0.29**	0	8	0.91	1.64	-3.36	1.59 (2.1)	4.82 (2.4)
4	PCL-M Cog/Mood				-	0.86**	− 0.00	0.06	0.01	− 0.29**	− 0.27**	− 0.27**	0	20	0.88	1.20	-2.84	4.99 (4.8)	12.12 (4.9)
5	PCL-M Arousal					-	− 0.01	0.06	0.08	− 0.29**	− 0.30**	− 0.28**	0	20	0.90	− 0.12	-2.82	6.44 (5.0)	13.38 (4.2)
6	span – Military						-	0.81**	0.76**	0.00	0.00	− 0.06	3	9	-	1.92	− 0.30	5.5 (1.3)	5.46 (1.3)
7	span – Negative							-	0.79**	0.00	0.05	− 0.08	3	9	-	2.55	0.68	5.36 (1.1)	5.56 (1.3)
8	span – Neutral								-	0.02	0.07	0.00	3	9	-	2.03	1.01	5.61 (1.2)	5.73 (1.4)
9	n-back – Military									-	0.89**	0.87**	− 0.73	4.65	-	0.16	-1.37	2.43 (1.3)	1.76 (1.2)
10	n-back– Negative										-	0.84**	-1.11	4.65	-	0.25	-1.12	2.38 (1.3)	1.77 (1.3)
11	n-back– Neutral											-	− 0.49	4.65	-	− 0.57	-1.32	2.32 (1.3)	1.87 (1.3)
	Study 2 (n = 211)	1	2	3	4	5	6	7					min	max	a	Sk	K	M (SD)	M (SD)
1	Age	-	0.15*	0.08	0.07	0.14*	0.10	0.06					19	78	-	2.12	− 0.24	42.46 (11.1)	46.97 (12.7)
2	PCL-M Intrusions		-	0.81**	0.77**	0.81**	0.20**	0.01					0	20	0.94	1.93	-3.45	4.11 (4.2)	11.09 (4.6)
3	PCL-M Avoidance			-	0.78**	0.75**	0.17*	− 0.02					0	8	0.88	1.32	-4.06	1.76 (2.2)	4.79 (2.3)
4	PCL-M Cog/Mood				-	0.85**	0.15*	− 0.04					0	20	0.89	0.73	-3.68	5.32 (5.1)	12.59 (4.8)
5	PCL-M Arousal					-	0.22**	0.03					0	20	0.90	− 0.19	-3.32	6.48 (4.8)	13.48 (4.4)
6	SUIS						-	0.54**					1	30	0.80	-1.24	-1.23	18.19 (5.7)	19.98 (5.6)
7	VVIQ							-					17	96	0.96	0.99	-2.09	60.89 (18.0)	60.59 (20.5)

Notes. PCL-M = PTSD ChecklistMilitary Version 5; VVIQ = Vividness of Visual Imagery Questionnaire; SUIS = Spontaneous Use of Imagery Scale; Sk = skewness statistic/ se; K = kurtosis statistic/ se; α = Cronbach’s alpha; *p < .05; **p < .01

The results of the logistic regression on self-reported PTSD diagnosis are summarized in Table 3 and reveal over 80% congruence between PCL-M subscale scores and participants’ self-described PTSD diagnosis history. We presume that the remaining 20% is attributable to a combination of measurement error, participants with a PTSD diagnosis whose symptoms eventually improved (perhaps in response to treatment), and/or the presence of undiagnosed participants in the non-PTSD group. Note that self-reported PTSD in combat personnel (45.9%) was not significantly lower than in non-combat personnel (39.6%), χ^2^(n = 138) = 0.52, *p* = .471ns. This supports the point made earlier that traumatic stress in the military context is not limited to combat exposure but can occur in relation to various moral injuries acquired during military service (e.g., Hall et al., 2022).

**Table 3:** Binary logistic regressions with PTSD diagnosis (1 = no, 2 = yes) regressed on PCL-M subscales

	Study 1 (N = 138)						Study 2 (N = 211)					
	model χ^2^ (df = 4)	*R* ^2^	B	SE	Wald ^2^ (df = 1)	Exp(B) [± 95% CI]	model χ^2^ (df = 4)	*R* ^2^	B	SE	Wald χ^2^ (df = 1)	Exp(B) [± 95% CI]
PCL-M – intrusions	67.45**	0.52	0.14	0.07	3.70*	1.15 [1.00-1.33]	104.82**	0.52	0.15	0.06	5.90*	1.16 [1.03–1.31]
PCL-M – avoidance			0.10	0.14	0.52	1.11 [0.84-1.46]			0.05	0.12	0.16	1.05 [0.83-1.32]
PCL-M – cog/mood			0.07	0.07	0.93	1.07 [0.93-1.24]			0.07	0.06	1.66	1.08 [0.96 − 1.20]
PCL-M – arousal			0.07	0.09	0.69	1.07 [0.91-1.27]			0.10	0.06	2.44	1.10 [0.98-1.25]
*Correct classification*	80.4%						80.6%					

Notes. PCL-M = PTSD Checklist – Military Version 5; PTSD diagnosis (1 = no diagnosis; 2 = PTSD diagnosis); Nagelkerke *R*^2^ estimates are reported; *p < .05; **p < .01

### Memory performance with affective interference

Descriptives and bivariate correlations in Table 2 confirm both sets of memory performance to be normally distributed and uncorrelated, with 2-back performance (but not memory span) negatively correlated with all four PTSD symptom clusters. The results of two ANCOVAs on memory performance (separately for memory span and 2-back) by PTSD diagnosis history and word type (military, emotional, neutral), with age included as a covariate, were used to evaluate hypotheses concerning WM differences as a function of PTSD diagnosis and the presence of task interference.

**Fig. 2:**
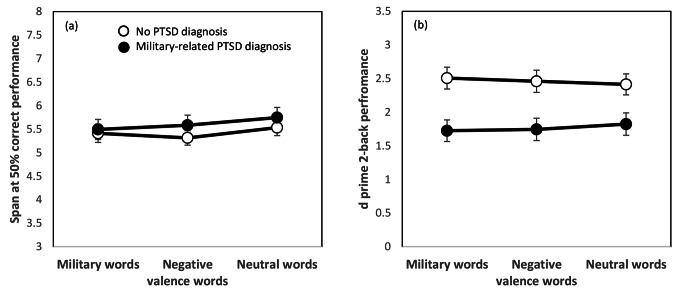
**Colour memory performance** as a function of affective distraction for non-PTSD and PTSD-diagnosed military personnel for Study 1 (N = 138). (a) Results for colour span task with performance given as sequence span at 50% correct performance. (b) Results for 2-back task with performance given as d prime values (d’ = z(Hits) - z(False alarms). ±1SE bars are included

The hypothesis that WM performance will be worse in military personnel diagnosed with PTSD (H1) was partly supported by a significant between-subjects main effect of PTSD group in relation to the 2-back task, *F*(1,122) = 9.71, *p* < .005, η^2^_p_ = 0.07, but not the memory span task, *F*(1,80) = 0.80, *p* = .373ns, η^2^_p_ = 0.01. As shown in Fig. 2b, this effect was in the expected direction, with PTSD-diagnosed participants performing worse on the 2-back compared to their non-PTSD peers.

The hypothesis that emotional and military-related words would interfere with memory performance, and that PTSD-diagnosed participants would be more vulnerable to this interference (H2), was not supported. First, no main effect of word-interference type was obtained either in relation to the working memory span task, *F*(2.160) = 0.35, *p* = .703ns, η^2^_p_ = 0.00, or the 2-back task, *F*(2,244) = 0.39, *p* = .676ns, η^2^_p_ = 0.00. That is, the type of word used to ‘carry’ color information – neutral, military-related, or negative-emotional – did not affect WM performance. Second, no interaction between word type and group was obtained, either for memory span *F*(2,160) = 0.51, *p* = .601ns, η^2^_p_ = 0.01, or 2-back performance, *F*(2,244) = 1.19, *p* = .305ns, η^2^_p_ = 0.01. That is, the failure of type of word to influence WM performance was observed both for PTSD-diagnosed participants and their non-PTSD peers.

### Memory performance in relation to PTSD symptom clusters

The absence of interference effects in relation to memory performance supported combining performance across word types to create two latent variables: ‘span’ and ‘n-back’. The structural model depicting paths from PCL-M subscales to these two latent variables was found to have adequate fit (see Fig. 1 caption) and was thus suitable for use in testing paths from PCL-M to memory performance. The hypothesis that PTSD symptom clusters would demonstrate differential relationships with memory performance (H3) was supported, with standardized regression weights showing that 2-back performance was negatively associated with intrusions (and accounted for approximately 18% of variance in 2-back performance), and that memory span was negatively associated with avoidance symptoms and positively associated with arousal systems (with approximately 8% of the variance in memory span attributable to the combination of the two symptom clusters).

## Discussion

The results revealed deficits in 2-back performance but not memory span in military personnel diagnosed with PTSD. This supports previous research indicating that symptoms of PTSD interfere with cognitive processes involved in information selection, inhibition, and updating (Blanchette & Caparos, 2016). However, we were not able to reproduce WM interference effects by introducing emotional content in the form of negative emotional or military-related words, nor could we replicate previous research showing that military personnel diagnosed with PTSD are more vulnerable to such interference (Larsen et al., 2019). This result suggests that deficits in 2-back performance observed in our study were unrelated to participants’ ability to inhibit task-irrelevant affective information or trauma-related information.

Using the four-factor PCL-M we were able to show that WM functioning was negatively related to PTSD-intrusions. Previously, it has been suggested that intrusions undermine WM because they interfere with inhibitory processes common to both emotional regulation strategies and WM (Mathew et al., 2022; Nejati et al., 2018). In the General Discussion, we interpret our findings as evidence that affective interference in PTSD is not due to impaired ability to inhibit task-irrelevant information, but due to the additional load on inhibitory processes posed by intrusive memories and emotions. In the following section we explore the possibility that this internal ‘noise’ is related to visual imagery.

## Study 2 – Visual imagery and PTSD symptomatology

The results of Study 1 indicated that PTSD-related intrusions are associated with impaired WM performance in military personnel. Intrusions are often experienced as unwanted and distressing visual memories or ‘flashbacks’ of military-related events, but they can also take the form of imagined, suspected, or feared visual ‘flashforwards’ (Reynolds & Brewin, 1998). It is possible that visual intrusions of this sort are processed by the visuospatial sketchpad of WM (Baddeley & Andrade, 2000), and interfere with its ability to maintain and manipulate spatial information during memory tasks. For example, it has been shown that visuospatial memory involved in completing a figure-copying task is impaired in people with a PTSD diagnosis (Gurvits et al., 2002). However, there is little research examining the relationship between visual imagery and PTSD symptom clusters that can support the involvement of visual intrusions. Furthermore, previous research in the area has tended to focus on the subjective vividness of visual imagery rather than the spontaneous use of visual imagery.

In Study 2, military personnel reported their PTSD diagnosis history and completed measures of PTSD symptomatology followed by two components of visual imagery – subjective vividness of imagery and spontaneous use of imagery in everyday tasks. The following hypothesis was tested:

H4: Visual imagery will be positively associated with PTSD diagnosis and symptom severity, particularly in relation to the intrusive PTSD symptoms.

## Method

### Participants

Participants were 211 military personnel or veterans between 19 and 75 years of age. Their personal characteristics are summarized in Table 1, showing them to be predominantly white Australian Army personnel who had deployed in a combat-related role.

### Procedure

The authors adhered to APA ethical standards in the recruitment and treatment of participants and the project was approved by our institution’s Human Research Ethics Committee. Ethical processes were as described in Study 1 and participants were recruited as per Study 1.

The study was in the form of a survey completed online via the Qualtrics™ survey system. All data were collected in a single session during the Covid-19 lockdown of 2021. Participants answered demographic questions (including questions about PTSD history) as per Study 1, then completed the following measures in the order in which they are described:

### Materials

#### PTSD checklist – military version (PCL‑M)

Details are as per Study 1. CFA of responses to the PCL-M again confirmed the four-factor structure proposed by the DSM 5, χ^2^(113) = 272.90, *p* < .05; χ^2^/df = 2.42, RMSEA = 0.082 [95% CI: 0.070-0.095], SRMR = 0.039, CFI = 0.95, and TLI = 0.94.

#### Spontaneous use of imagery scale (SUIS)

Participants used 5-point Likert-type scales anchored from 1 (it is never appropriate) to 5 (it is always completely appropriate) to respond to 12 statements describing their spontaneous/general use of visual mental images when performing everyday tasks (Nelis et al., 2014), such as: “*If someone were to tell me two-digit numbers to add (e.g., 24 and 31), I would visualize them in order to add them.*” Higher scores on the measure indicate more frequent use of visual imagery. The measure has acceptable reliability and convergent validity (Nelis et al., 2014).

#### Vividness of mental imagery questionnaire (VVIQ)

Participants were asked to visualize four commonplace events (e.g., a sunrise) and use 5-point Likert-type scales anchored between 1 (no image at all) and 5 (perfectly clear and as vivid as normal vision) to respond to a total of 16 questions about the vividness of the images they experience as they imagine the event in various ways, such as: “*The sky clears and surrounds the sun with blueness.*” Higher scores on the measure indicate more vivid visual imagery (note that we reverse-coded the responses compared to the original measure to make the direction of these scales consistent with the other two questionnaires). The measure is widely used and has acceptable reliability and validity (Marks, 1973, 1995).

### Data scoring procedures

As in Study 1, survey items were subject to response validation, thus ensuring that there were no missing values. Initial CFAs were conducted on the four-factor PCL-M and the unidimensional SUIS and VVIQ. Improvements to model fit were considered by removing non-significant or low factor loadings (< 0.5), and including correlated errors where indicated by modification indices. If final fits were confirmed, subscale scores could be computed and screened for normality on the basis of skew and kurtosis. If CFAs failed, exploratory factor analyses (EFAs) would be used to identify an alternative factor structure suitable for use in inferential analyses. EFAs would be conducted in two stages – a principal components analysis using Promax rotation (kappa = 4) to identify the number of factors with Eigenvalues > 1, followed by principal axis factoring with Promax rotation on these factors (Matsunaga, 2010).

### Data analysis

**Fig. 3:**
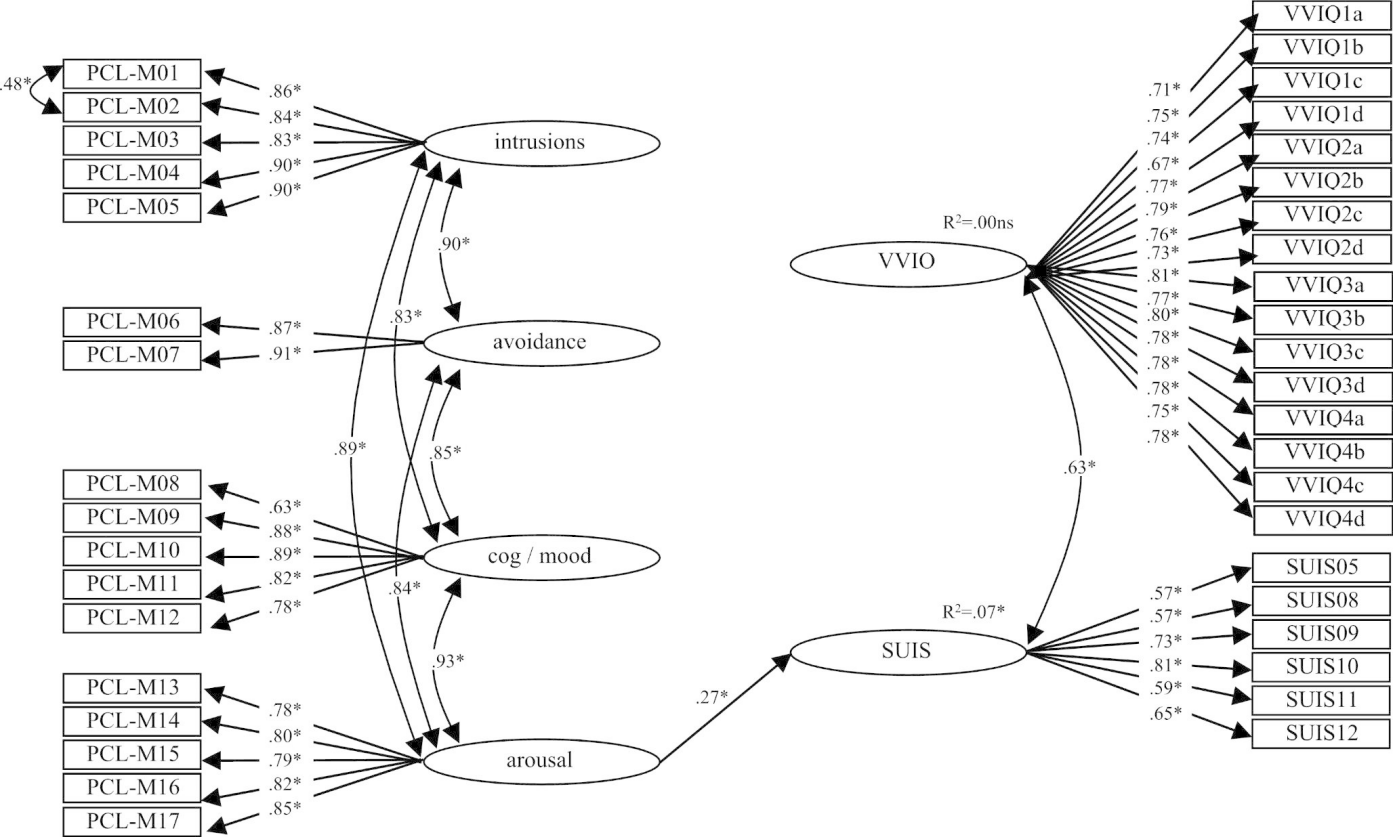
**Structural model for Study 2** (model optimized by removal of non-significant paths) with standardized statistics included. Model fit: χ^2^(685) = 1326.16, *p* < .001; χ^2^/df = 1.94; RMSEA = 0.067, 90% CI [0.061, 0.072]; SRMR = 0.057; CFI = 0.91, and TLI = 0.91. *p < .05; N = 211

As in Study 1, self-reported PTSD diagnosis history was validated against PCL-M subscales. Two between-groups t-tests were then conducted to test whether PTSD-diagnosed military personnel reported more vivid and/or more spontaneous use of visual imagery, and the structural model depicted in Fig. 3 was used to test paths from PTSD symptom clusters to the visual imagery variables identified previously in CFAs.

## Results

### Factorial structure of the PCL-M, SUIS, and VVIQ

As in Study 1, the binary logistic regression of PTSD diagnosis (1 = not diagnosed; 2 = diagnosed) by PCL-M subscales (see results in Table 3) validated our measure of PTSD diagnosis, with 80% correct classification of participants into PTSD/non-PTSD groups. As shown in Study 1, self-reported PTSD diagnoses were not significantly higher in combat personnel (54.8%) than in non-combat personnel (53.6%), χ^2^(n = 211) = 0.02, *p* = .871ns, again supporting the contribution of non-combat stressors to PTSD in the military context.

The original unidimensional SUIS model did not meet fit criteria. EFA supported the original unidimensional model but only after the removal of items 1–4 and 6–7 for having low and/or crossed factor loadings according to the ‘.6/.3’ rule (Matsunaga, 2010) and/or having low item-total correlations (< 0.2). CFA results on this highly modified model indicate acceptable fit, c^2^(9) = 19.77, *p* < .05, c^2^/df = 2.20, RMSEA = 0.075 [95% CI: 0.029-0.121], SRMR = 0.037, CFI = 0.97, and TLI = 0.95. The unidimensional VVIQ model also produced questionable results, χ^2^(96) = 462.76, *p* < .05; χ^2^/df = 4.82, RMSEA = 0.135 [95% CI: 0.123-0.147], SRMR = 0.070, CFI = 0.89, and TLI = 0.86. However, item-total correlations were good (all > 0.7), and Cronbach’s alpha was high (0.96). Moreover, EFAs did not reveal the presence of a superior primary-order factor structure. Therefore, despite some reservations, CFAs supported the computation of four PCL-M subscale scores, a single-factor SUIS, and single-factor VVIQ. These variables are summarized in Table 2 and confirm the presence of acceptable normality as well as high intercorrelations between PCL-M subscales.

### Visual imagery and PTSD diagnosis and symptomatology

Separate between-groups t-tests conducted on vividness of visual imagery and spontaneous use of visual imagery by military-related PTSD diagnosis (H4) indicated no group difference in relation to VVIQ (vividness of visual imagery), *t*(209) = 0.11, *p* = .91ns, but significantly higher SUIS ratings (spontaneous use of visual imagery) in PTSD-diagnosed personnel (M = 19.98, SD = 5.6) compared to non-PTSD personnel (M = 18.19, SD = 5.7), *t*(209) = 2.29, *p* < .05. The structural model depicted in Fig. 3 was confirmed to be adequate (see caption of Fig. 3) for the purposes of testing relationships between PTSD and visual imagery. The hypothesis that the PTSD symptom clusters would demonstrate differential interference effects on imagery ratings (H4) was supported but only in relation to SUIS scores. Inspection of standardized regression weights in Fig. 3 shows that frequency of spontaneous imagery was positively associated with arousal (this accounted for approximately 7% of variance in imagery). There were no significant paths from the PCL-M subscales to VVIQ.

## Discussion

It was hypothesized that intrusions would manifest as visual flashbacks (Bryant & Harvey, 1996). This was not supported in relation to vividness of visual imagery, however, military personnel diagnosed with PTSD reported experiencing more spontaneous visual imagery than their non-PTSD peers, particularly in relation to arousal symptoms of PTSD. Together, these results suggest that arousal does not trigger flashbacks in the form of autobiographical memories of trauma, but flashforwards about imagined/feared threats (Holmes et al., 2007; Rachman & de Silva, 1978). We explore this idea further in the following section.

## General discussion

Two studies investigated the relevance of PTSD for visual memory and visual imagery in military personnel. In Study 1 we looked for evidence that PTSD symptoms interfere with memory functioning, particularly the ability to inhibit task-irrelevant content. In Study 2 we looked for evidence these symptoms are related to the experience of visual imagery.

### Evidence of working memory deficits in PTSD

In Study 1 we observed that 2-back performance was impaired in PTSD-diagnosed military personnel. This replicated previous findings pointing to memory deficits in this population (Nejati et al., 2018; Shipstead et al., 2012). To understand why, consider that in each step of a 2-back sequence a participant is required not only to update the location in memory of a stored target within a sequence (Baddeley, 1986), but also to inhibit out-of-date information from the previous location in the sequence (Larsen et al., 2019). This suggests at least three possible ways in which PTSD symptoms might disrupt n-back performance, the most basic of which is by interfering with the ability to store information in memory in the first place. However, we found that memory span performance in our study was spared, suggesting that PTSD deficits are limited to WM functioning and do not generalize to memory capacity.

A second possibility is that PTSD symptoms increase *external* noise (such as the task-irrelevant affective and trauma-related distractor words used in Study 1) that WM processes are required to inhibit (Mathew et al., 2022). For example, PTSD-arousal symptoms are thought to cause and be caused by negative interpretive biases when facing potential threats, particularly in contexts that resemble the original traumatic event (Armour et al., 2017; Bomyea et al., 2017), and also negative flashforwards about imagined threats (Holmes et al., 2007). These negative influences have been shown to interfere with the inhibition of negative emotional content and responses to this content (Albanese et al., 2021; Ling et al., 2022). However, we did not find evidence to support the idea that PTSD contributes external noise sufficient to disrupt WM functioning. We found no impairments in the ability of participants diagnosed with PTSD to inhibit negative emotional or military-related distractors, nor did we observe relationships between arousal symptoms of PTSD and n-back performance more generally.

A third possibility is that PTSD symptoms contribute *internal* noise in the form of intrusive memories and emotions that interfere with the functioning of WM inhibitory processes. This leads to an accumulation of task-irrelevant information in stored memory as the participant works their way through the n-back task, and/or contributes additional task-irrelevant content that further taxes these inhibitory processes. According to this idea, a person suffering from intrusions would have to inhibit *external* task-irrelevant information (contained in the n-back task itself and also introduced by us in the form of distractors) while also contending with *internal* task-irrelevant information in the form of flashbacks. In support of this third option, we found that intrusive PTSD symptoms were indeed significantly (and negatively) associated with 2-back performance.

Finally, although group differences in working memory span were not observed, we did find evidence of slight negative relationships between PTSD avoidance and memory span consistent with the idea that trauma-avoidance strategies have wide-ranging impact on cognitive performance (Mathew et al., 2022), as well as an unexpected positive relationship between PTSD arousal and memory span. Outside of the PTSD context, there is evidence that moderate levels of psychophysiological arousal can improve some cognitive functions, including memory span (Castellà et al., 2020). These improvements are thought to arise in part because arousal can increase selective attention to a stimulus, its location, or the task more generally (Vaughn et al., 2021). In this context, our finding suggests that psychophysiological arousal can confer performance benefits even when the arousal originates from a presumably distressing event.

### Evidence of altered visual imagery in PTSD

In Study 2, PTSD-diagnosed military personnel reported experiencing more spontaneous visual imagery in their everyday lives. This result is consistent with the idea that the visuospatial sketchpad is more active in people with PTSD (Keogh & Pearson, 2014). Autobiographical memories of a traumatic event can be very vivid and emotionally disruptive (Bryant & Harvey, 1996; Thome et al., 2020), and in our study were hypothesized to be the likely source of this visual imagery. However, we found no evidence for this in the results of Study 2, with imagery vividness ratings shown to be similar between PTSD and non-PTSD participants, and unrelated to any PTSD symptoms, including intrusions.

Interestingly, spontaneous visual imagery was related to PTSD symptoms, but only symptoms of arousal. That visual imagery was linked to arousal, but not intrusions, is more consistent with flashforwards about imagined future threats rather than with flashbacks of past threats (Reynolds & Brewin, 1998). Flashforwards have been reported previously in trauma-related conditions as well as in a range of affective conditions such as depression and obsessive-compulsive disorder (Engelhard et al., 2011). Unlike intrusions in the form of flashbacks about the original event (Strohm et al., 2021), flashforwards are thought to exacerbate and maintain PTSD symptoms by causing a focus on fear and anxiety responses as well as lowering motivation for positive change (Landkroon et al., 2022).

### Limitations

A limitation of the research was its reliance on self-report measures. Although their use was convenient and allowed for a larger and broader participant sample, especially during the Covid-19 pandemic, it also meant that our measure of PTSD diagnosis was not independently verified using a clinician-administered PTSD scale (Kok et al., 2012), but relied instead on verification against current PTSD symptoms measured using a self-administered screening tool. Fortunately, this approach did demonstrate agreement between self-reported PTSD diagnosis history and PTSD symptomatology. The inclusion of symptomatology in analyses also provided a useful dimensional perspective to our understanding of the relationship between PTSD and cognitive functioning (cf. Mota et al., 2016), one that is consistent with longitudinal evidence that even subclinical levels of PTSD in military personnel are predictive of future PTSD diagnosis (Highfill-McRoy et al., 2022) as well as a range of psychosocial difficulties (Fink et al., 2018).

There were similar concerns with the VVIQ measure of visual imagery commonly used in imagery research. This measure not only relies on self-report, it targets subjective components of imagery that cannot be validated against objective criteria. We addressed this limitation in Study 2 by including a measure of imagery that assesses frequency of use of visual imagery, and it was this component of visual imagery, rather than imagery vividness, that was found to be relevant to PTSD diagnosis and symptomatology.

Our research also relied on cross-sectional research designs and correlational analyses making it impossible for us to draw casual inferences from the results. This was limiting particularly in relation to understanding the impact of PTSD symptoms on memory and imagery where symptoms (e.g., intrusive memories and imagined fears) have features in common with the cognitive function being measured. As is evident in Table 1, numerous participants were also medicated at the time of completing the study. This could have influenced their cognitive functioning and/or reduced the severity of PTSD and its effects on their cognitive performance. Finally, we relied on changing the semantic context of words to introduce emotional and military-related task-irrelevant interference (Ben-Haim et al., 2016). It may be that the words we chose were insufficiently salient to produce a measurable degradation of WM performance in military personnel.

### Practical implications

Before concluding, it is worth considering the practical implications of our findings. In replicating findings that WM is impaired in military personnel with a PTSD diagnosis or in relation to PTSD symptomatology, our results provide general support for cognitive training interventions for PTSD, particularly those based on n-back tasks (Larsen et al., 2019), that seek to improve cognitive control and attentional focus in military personnel (McDermott et al., 2016). The associations we observed between WM performance and PTSD symptoms of intrusions and arousal are also consistent with the inclusion of training components designed to improve emotional regulation in trauma survivors (e.g., Barkus, 2020). However, in finding no evidence of greater vulnerability to trauma-related or negative emotional interference in this population, our results argue against the idea that such interventions would benefit by the addition of trauma-related or emotional contexts (e.g., Schweizer et al., 2013). Finally, although there has been less interest in addressing imagery-related symptoms of PTSD, such as flashforwards, our finding that utilization of visual imagery was associated with PTSD/PTSS, supports continued research into therapies, such as imagery rehearsal therapy, that involves reimagining disturbing recurring thoughts and dreams in trauma survivors for therapeutic purposes (Belleville et al., 2018), and also supports continuing work into methods of altering WM processes (e.g., using eye movements to tax WM resources) to reduce unwanted flashforwards (Engelhard et al., 2011).

## Conclusion

Although we confirmed that WM performance is worse in military personnel diagnosed with PTSD as well as in those with elevated PTSD symptomatology, we found no evidence of their greater sensitivity to emotional or military-related distractors. This argues against the idea that PTSD interferes with inhibitory WM processes. We observed instead that intrusive symptoms of PTSD were associated with poorer WM performance, suggesting that WM is impaired not because of problems with inhibitory processes per se, but because intrusive flashbacks add internal noise that taxes these processes. In terms of the role of PTSD in visual imagery, we found no evidence to suggest that intrusions are associated with more vivid or more frequent experiences of visual imagery. However, arousal symptoms were associated with more visual imagery, perhaps in the form of flashforwards about feared/anticipated threats. These results demonstrate the benefits of a closer examination of associations involving PTSD at the level of individual symptom clusters.

## Data Availability

The study reported in the article was not formally preregistered. Neither the data nor the materials have been made available on a permanent third-party archive; requests for the data or materials should be sent via email to the corresponding author.

## References

[CR1] Ahsen A (1995). Self-report questionnaires: new directions for imagery research. Journal of Mental Imagery.

[CR2] Albanese BJ, Preston TJ, Bedford C, Macatee RJ, Schmidt NB (2021). Distress intolerance prospectively predicts traumatic intrusions following an experimental trauma in a non-clinical sample. Cognitive Therapy & Research.

[CR3] American Psychiatric Association (2022). *Diagnostic and Statistical Manual of Mental Disorders* (fifth edition, text revision ed.). American Psychiatric Association.

[CR4] Armenta RF, Walter KH, Geronimo-Hara TR, Porter B, Stander VA, LeardMann CA (2019). Longitudinal trajectories of comorbid PTSD and depression symptoms among U.S. service members and veterans. Bmc Psychiatry.

[CR5] Armour C, Fried EI, Deserno MK, Tsai J, Pietrzak RH (2017). A network analysis of DSM-5 posttraumatic stress disorder symptoms and correlates in U.S. military veterans. Journal of Anxiety Disorders.

[CR6] Atkinson, R. C., & Shiffrin, R. M. (1971). *The control processes of short-term memory*. Institute for Mathematical Studies in the Social Sciences.

[CR7] Baddeley, A. D. (1986). *Working Memory*. Oxford University Press.

[CR8] Baddeley AD (2002). Is working memory still working?. European Psychologist.

[CR9] Baddeley AD, Andrade J (2000). Working memory and the vividness of imagery. Journal of Experimental Psychology: General.

[CR10] Barkus E (2020). Effects of working memory training on emotion regulation: transdiagnostic review. PsyCh Journal.

[CR11] Belleville G, Dubé-Frenette M, Rousseau A, Dubé-Frenette M (2018). Efficacy of Imagery Rehearsal therapy and cognitive behavioral therapy in sexual assault victims with posttraumatic stress disorder: a Randomized Controlled Trial. Journal of Traumatic Stress.

[CR12] Ben-Haim, M. S., Williams, P., Howard, Z., Mama, Y., Eidels, A., & Algom, D. (2016). The emotional Stroop task: assessing cognitive performance under exposure to emotional content. *Journal of Visualized Experiments: JoVE*, (112). 10.3791/53720 10.3791/53720 PMC4993290 27405091

[CR13] Berger, N., Richards, A., & Davelaar, E. J. (2017). When emotions matter: focusing on emotion improves working memory updating in older adults. *Frontiers in Psychology*, *8*. 10.3389/fpsyg.2017.01565 10.3389/fpsyg.2017.01565 PMC5605649 28966602

[CR14] Blanchette I, Caparos S (2016). Working memory function is linked to trauma exposure, independently of post-traumatic stress disorder symptoms. Cognitive Neuropsychiatry.

[CR15] Boffa JW, Norr AM, Tock JL, Amir N, Schmidt NB (2018). Development of the interpretation bias index for PTSD. Cognitive Therapy and Research.

[CR16] Bomyea J, Johnson A, Lang AJ (2017). Information processing in PTSD: evidence for biased attentional, interpretation, and memory processes. Psychopathology Review.

[CR17] Brewin CR, Dalgleish T, Joseph S (1996). A dual representation theory of posttraumatic stress disorder. Psychological Review.

[CR18] Brownlow JA, Zitnik GA, McLean CP, Gehrman PR (2018). The influence of deployment stress and life stress on post-traumatic stress disorder (PTSD) diagnosis among military personnel. Journal of Psychiatric Research.

[CR19] Bryant RA, Harvey AG (1996). Visual imagery in posttraumatic stress disorder. Journal of Traumatic Stress.

[CR20] Castellà J, Boned J, Méndez-Ulrich JL, Sanz A (2020). Jump and free fall! Memory, attention, and decision-making processes in an extreme sport. Cognition & Emotion.

[CR21] Dance CJ, Ipser A, Simner J (2022). The prevalence of aphantasia (imagery weakness) in the general population. Consciousness and Cognition.

[CR22] Daneshvar S, Taghavi MR, Jobson L (2021). Proactive interference in posttraumatic stress disorder. Journal of Traumatic Stress.

[CR23] Engelhard IM, van den Hout MA, Dek EC, Giele CL, van der Wielen JW, Reijnen MJ, van Roij B (2011). Reducing vividness and emotional intensity of recurrent “flashforwards” by taxing working memory: an analogue study. Journal of Anxiety Disorders.

[CR24] Erickson LD, Hedges DW, Call VR, Bair B (2013). Prevalence of and factors associated with subclinical posttraumatic stress symptoms and PTSD in urban and rural areas of Montana: a cross-sectional study. The Journal of Rural Health.

[CR25] Fink DS, Gradus JL, Keyes KM, Calabrese JR, Liberzon I, Tamburrino MB, Cohen GH, Sampson L, Galea S (2018). Subthreshold PTSD and PTSD in a prospective-longitudinal cohort of military personnel: potential targets for preventive interventions. Depression and Anxiety.

[CR26] Gerber MM, Frankfurt SB, Contractor AA, Oudshoorn K, Dranger P, Brown LA (2018). Influence of multiple traumatic event types on mental health outcomes: does count matter?. Journal of Psychopathology and Behavioral Assessment.

[CR27] Gurvits TV, Lasko NB, Repak AL, Metzger LJ, Orr SP, Pitman RK (2002). Performance on visuospatial copying tasks in individuals with chronic posttraumatic stress disorder. Psychiatry Research.

[CR28] Hall NA, Everson AT, Billingsley MR, Miller MB (2022). Moral injury, mental health and behavioural health outcomes: a systematic review of the literature. Clinical Psychology & Psychotherapy.

[CR29] Highfill-McRoy, R. M., Levine, J. A., Larson, G. E., Norman, S. B., Schmied, E. A., & Thomsen, C. J. (2022). Predictors of symptom increase in subsyndromal PTSD among previously deployed military personnel. *Military Medicine*, *187*(5–6). 10.1093/milmed/usab034. e711-e717. 10.1093/milmed/usab034 PMC9071097 33580699

[CR30] Holmes EA, Crane C, Fennell MJ, Williams JMG (2007). Imagery about suicide in depression—“Flash-forwards”?. Journal of Behavior Therapy and Experimental Psychiatry.

[CR31] Hu L, Bentler PM (1999). Cutoff criteria for fit indexes in covariance structure analysis: conventional criteria versus new alternatives. Structural Equation Modeling: A Multidisciplinary Journal.

[CR32] Hunston S (2002). Word frequencies in written and spoken English: based on the British National Corpus. Language Awareness.

[CR33] Judah MR, Renfroe JB, Wangelin BC, Turner TH, Tuerk PW (2018). Hyperarousal symptoms explain the relationship between cognitive complaints and working memory performance in veterans seeking PTSD treatment. The Journal of Head Trauma Rehabilitation.

[CR34] Kaimal G, Walker MS, Herres J, Berberian M, DeGraba TJ (2022). Examining associations between montage painting imagery and symptoms of depression and posttraumatic stress among active-duty military service members. Psychology of Aesthetics Creativity and the Arts.

[CR35] Kane MJ, Engle RW (2003). Working-memory capacity and the control of attention: the contributions of goal neglect, response competition, and task set to Stroop interference. Journal of Experimental Psychology: General.

[CR36] Keogh, R., & Pearson, J. (2014). The sensory strength of voluntary visual imagery predicts visual working memory capacity. *Journal of Vision*, *14*(12). 10.1167/14.12.7 25301015

[CR37] Keogh R, Wicken M, Pearson J (2021). Visual working memory in aphantasia: retained accuracy and capacity with a different strategy. Cortex; A Journal Devoted To The Study Of The Nervous System And Behavior.

[CR38] Kessler RC, Aguilar-Gaxiola S, Alonso J, Benjet C, Bromet EJ, Cardoso G, Degenhardt L, de Girolamo G, Dinolova RV, Ferry F (2017). Trauma and PTSD in the WHO world mental health surveys. European Journal of Psychotraumatology.

[CR39] Knobloch LK, Owens JL, Gobin RL (2022). Soul wounds among combat trauma survivors: experience, effects, and advice. Traumatology.

[CR40] Kok BC, Herrell RK, Thomas JL, Hoge CW (2012). Posttraumatic stress disorder associated with combat service in Iraq or Afghanistan: reconciling prevalence differences between studies. Journal of Nervous and Mental Disease.

[CR41] Landkroon E, Meyerbröker K, Salemink E, Engelhard IM (2022). Future-oriented imagery rescripting facilitates conducting behavioral experiments in social anxiety. Behaviour Research and Therapy.

[CR42] Larsen SE, Lotfi S, Bennett KP, Larson CL, Dean-Bernhoft C, Lee HJ (2019). A pilot randomized trial of a dual n-back emotional working memory training program for veterans with elevated PTSD symptoms. Psychiatry Research.

[CR43] Lee DJ, Lee LO, Bovin MJ, Moshier SJ, Dutra SJ, Kleiman SE, Rosen RC, Vasterling JJ, Keane TM, Marx BP (2020). The 20-year course of posttraumatic stress disorder symptoms among veterans. Journal of Abnormal Psychology.

[CR44] Ling J, Keegan FS, Weiss NH, Alghraibeh AM, Aljomaa SS, Almuhayshir AR, Contractor AA (2022). Examining indirect effects of emotion dysregulation between PTSD symptom clusters and reckless/self-destructive behaviors. Psychological Trauma: Theory Research Practice & Policy.

[CR45] Litz BT, Keane TM (1989). Information processing in anxiety disorders: application to the understanding of post-traumatic stress disorder. Clinical Psychology Review.

[CR46] Litz BT, Stein N, Delaney E, Lebowitz L, Nash WP, Silva C, Maguen S (2009). Moral injury and moral repair in war veterans: a preliminary model and intervention strategy. Clinical Psychology Review.

[CR47] Marks DF (1973). Visual imagery differences in the recall of pictures. British Journal of Psychology.

[CR48] Marks DF (1995). New directions for mental imagery research. Journal of Mental Imagery.

[CR49] Mathew AS, Lotfi S, Bennett KP, Larsen SE, Dean C, Larson CL, Lee HJ (2022). Association between spatial working memory and re-experiencing symptoms in PTSD. Journal of Behavior Therapy and Experimental Psychiatry.

[CR50] Matsunaga M (2010).

[CR51] McDermott T, Badura-Brack A, Becker K, Ryan T, Bar-Haim Y, Pine D, Khanna M, Heinrichs-Graham E, Wilson T (2016). Attention training improves aberrant neural dynamics during working memory processing in veterans with PTSD. Cognitive Affective & Behavioral Neuroscience.

[CR52] Morina N, Leibold E, Ehring T (2013). Vividness of general mental imagery is associated with the occurrence of intrusive memories. Journal of Behavior Therapy and Experimental Psychiatry.

[CR53] Mota NP, Schaumberg K, Vinci C, Sippel LM, Jackson M, Schumacher JA, Coffey SF (2015). Imagery vividness ratings during exposure treatment for posttraumatic stress disorder as a predictor of treatment outcome. Behaviour Research & Therapy.

[CR54] Mota NP, Tsai J, Sareen J, Marx BP, Wisco BE, Harpaz-Rotem I, Southwick SM, Krystal JH, Pietrzak RH (2016). High burden of subthreshold DSM-5 post-traumatic stress disorder in U.S. military veterans. World Psychiatry.

[CR55] Neil, A. M., & Creelman, C. D. (2005). *Detection theory: a user’s guide* (2Vol. nd ed.). Psychology Press.

[CR56] Nejati V, Salehinejad MA, Sabayee A (2018). Impaired working memory updating affects memory for emotional and non-emotional materials the same way: evidence from post-traumatic stress disorder (PTSD). Cognitive Processing.

[CR57] Nelis S, Holmes EA, Griffith JW, Raes F (2014). Mental imagery during daily life: psychometric evaluation of the spontaneous use of Imagery Scale (SUIS). Psychologica Belgica.

[CR58] Nordstrand AE, Bøe HJ, Holen A, Reichelt JG, Gjerstad CL, Hjemdal O (2019). Danger- and non-danger-based stressors and their relations to posttraumatic deprecation or growth in norwegian veterans deployed to Afghanistan. European Journal of Psychotraumatology.

[CR59] Rachman S, de Silva P (1978). Abnormal and normal obsessions. Behaviour Research and Therapy.

[CR60] Rączy K, Orzechowski J (2021). When working memory is in a mood: combined effects of induced affect and processing of emotional words. Current Psychology: A Journal for Diverse Perspectives on Diverse Psychological Issues.

[CR61] Reger GM, Bourassa KJ, Smolenski D, Buck B, Norr AM (2019). Lifetime trauma exposure among those with combat-related PTSD: Psychiatric risk among US Military personnel. Psychiatry Research.

[CR62] Reynolds M, Brewin CR (1998). Intrusive cognitions, coping strategies and emotional responses in depression, post-traumatic stress disorder and a non-clinical population. Behaviour Research and Therapy.

[CR63] Ribeiro FS, Santos FH, Albuquerque PB (2019). How does allocation of emotional stimuli impact working memory tasks? An overview. Advances in Cognitive Psychology.

[CR64] Schweizer S, Gotlib IH, Blakemore SJ (2020). The role of affective control in emotion regulation during adolescence. Emotion.

[CR65] Schweizer S, Grahn J, Hampshire A, Mobbs D, Dalgleish T (2013). Training the emotional brain: improving affective control through emotional working memory training. The Journal of Neuroscience.

[CR66] Shipstead Z, Redick TS, Engle RW (2012). Is working memory training effective?. Psychological bulletin.

[CR67] Strohm M, Siegesleitner M, Kunze AE, Werner GG, Ehring T, Wittekind CE (2021). Psychological and physiological effects of imagery rescripting for aversive autobiographical memories. Cognitive Therapy & Research.

[CR68] Tas AC, Costello MC, Buss AT (2020). Age-related decline in visual working memory: the effect of nontarget objects during a delayed estimation task. Psychology and Aging.

[CR69] Thome J, Terpou BA, McKinnon MC, Lanius RA (2020). The neural correlates of trauma-related autobiographical memory in posttraumatic stress disorder: a meta‐analysis. Depression and Anxiety.

[CR70] VanBergen A, Blalock J, Bryant A, Bortz P, Bartle-Haring S (2020). Couples and trauma history: a descriptive overview of interpersonal trauma and clinical outcomes. Contemporary Family Therapy: An International Journal.

[CR71] Vaughn, D. A., Maggiora, M. B., Vaughn, K. J., Maggiora, C. J., Tavakoli, A. V., Liang, W., Zava, D., Cohen, M. S., & Lenartowicz, A. (2021). Modulation of attention and stress with arousal: the mental and physical effects of riding a motorcycle. *Brain Research*, *1752*(1). 10.1016/j.brainres.2020.147203 10.1016/j.brainres.2020.147203 33482998

